# Androgen receptor gene polymorphisms and risk of prostate cancer: a meta-analysis

**DOI:** 10.1038/srep40554

**Published:** 2017-01-16

**Authors:** Hong Weng, Sheng Li, Jing-Yu Huang, Zi-Qi He, Xiang-Yu Meng, Yue Cao, Cheng Fang, Xian-Tao Zeng

**Affiliations:** 1 Center for Evidence-Based and Translational Medicine, Zhongnan Hospital of Wuhan University, Wuhan 430071, P.R. China; 2 Center for Evidence-Based and Translational Medicine, Wuhan University, Wuhan 430071, P.R. China; 3 Department of Urology, Zhongnan Hospital of Wuhan University, Wuhan 430071, P.R. China; 4 Department of Urology, First Affiliated Hospital of Guangxi Medical University, Nanning 530021, P.R. China

## Abstract

Although the association between CAG and GGN repeats in the androgen receptor gene and prostate cancer risk has been widely studied, it remains controversial from previous meta-analyses and narrative reviews. Therefore, we performed this meta-analysis to provide more precise estimates with sufficient power. A total of 51 publications with 61 studies for CAG repeats and 14 publications with 16 studies for GGN repeats were identified in the meta-analysis. The results showed that short CAG repeats (<22 repeats) carriers presented an elevated risk of prostate cancer than long CAG repeats (≥22) carriers (OR = 1.31, 95% CI 1.16 to 1.47). Prostate cancer cases presented an average fewer CAG repeats (MD = −0.85, 95% CI −1.28 to −0.42) than controls. Short GGN repeats (≤16) carriers presented an increased risk of prostate cancer than long GGN repeats (>16) carriers (OR = 1.38, 95% CI 1.05 to 1.82). In subgroup analyses, the abovementioned significant association was predominantly observed in Caucasian populations. The meta-analysis showed that short CAG and GGN repeats in androgen receptor gene were associated with increased risk of prostate cancer, especially in Caucasians.

Prostate cancer ([Mendelian Inheritance in Man 176807]) is the most commonly diagnosed nonskin malignancy and the second leading cause of cancer-related death among men in United States and first leading cause of death among Hispanics/Latinos^1^,^2^; and in Asian countries, especially in China, the incidence of prostate cancer is increasing^3^. Worldwide, the disease is the second most common cancer in men after lung cancer^4^. Prostate cancer is a complicated and multifactorial disease. The precise etiology and pathological mechanism of prostate cancer remains unclear. Age, family history, and ethnicity are the most consistently addressed risk factors associated with prostate cancer. However, age and inherited factors are estimated to be responsible for 5% to 9% percentage of prostate cancer^5^. Therefore, identifying a preventable cause of prostate cancer would produce an important influence of public health.

The substantial differences aforementioned in incidence of prostate cancer worldwide may be due to ethnic variation^6^. Therefore, certain researchers indicated that different levels of androgens across varying ethnicity may contribute to these differences^6^,^7^. The exact mechanism through which androgen is involved in the etiology of prostate cancer remains unclear. The androgen receptor gene [Mendelian Inheritance in Man 313700] is located at Xq11.2-q12, and the length of androgen receptor gene is more than 90 kb^8^. The androgen receptor gene is comprised of eight exons that encode four functional domains, which include the transactivation domain, the DNA binding domain, a hinge region, and the carboxyl-terminal ligand binding domain^9^. There are two main polymorphisms including CAG and GGN repeats in the androgen receptor gene. Moreover, CAG was associated with the transcriptional activity of the AR in response to ligand binding. Therefore, the correlation between these polymorphisms of androgen receptor and risk of prostate cancer has received much attention. Three published meta-analyses^6^,^10^,^11^ and several narrative reviews^12^,^13^,^14^,^15^ have addressed the association between the repeat polymorphisms and prostate cancer susceptibility. However, the conclusions of these previous meta-analyses were not consistent and the narrative reviews could not quantify the estimate. Additionally, more studies have been published since the most recent meta-analysis. Therefore, we performed the present meta-analysis aimed to provide a more precise and comprehensive result for the relationship between CAG and GGN repeat polymorphisms of androgen receptor gene and prostate cancer susceptibility.

## Methods

### Eligible criteria

For inclusion in this meta-analysis, the publication had to meet the following eligible criteria: (1) the exposure was androgen receptor gene CAG and GGN repeat polymorphisms; (2) populations were men with prostate cancer (cases) without prostate cancer (controls); (3) the outcome was incident of prostate cancer; (4) the study design was retrospective or prospective (i.e. nested) case-control study; (5) study provided distribution of genotype, odds ratio (OR) and corresponding 95% confidence interval (CI), mean difference (MD) and corresponding standard error (SE), and mean repeats in case and control groups with related SE. For duplicated publication, we included the most recent or that providing the most information. If one publication provided different groups of ethnicity, we considered the each group as a separate study. We conducted the meta-analysis according to Preferred Reporting Items for Systematic Reviews and Meta-Analyses (PRISMA) statement in reporting meta-analysis. The protocol (registration number: CRD42016036971) of the meta-analysis was published in the PROSPERO register (http://www.crd.york.ac.uk/PROSPERO/).

### Search strategy

A comprehensively literature search was performed in PubMed, Embase, CBM, CNKI and Wanfang databases up to March, 2016, without restriction to regions, publication types, or languages. The search strategy was as following: (“polymorphism” AND “prostate cancer” AND “androgen receptor”). In addition, references in the recent reviews or meta-analysis and included articles were identified for any further potential related studies.

### Data extraction

Data from the included studies were extracted and summarized independently by two authors (HW and XT-Z). Any disagreement was resolved by discussion of which data should be extracted. The following information was extracted: last name of first author, publication year, country of study, ethnicity, study design, control status, sample size, age of the cases and controls, percentage of advanced prostate cancer cases (T3-T4, M0; T0-T4, M1), the repeat cutpoint of polymorphisms, mean number of repeats in case and control groups with related SE, dichotomous data (short versus long repeats), and estimate with corresponding 95% CI (including OR for dichotomous data and continuous data). We defined the long CAG and GGN repeats as ≥22 and >16 repeats as previously published^6^, respectively. Otherwise, <22 and ≤16 were short CAG and GGC repeats, respectively.

### Statistical analysis

We calculated ORs and 95% CIs for short CAG repeats (<22) versus long CAG repeats (≥22) and short GGN repeats (≤16) versus long GGN repeats (>16) using dichotomous data^6^. We summarized ORs and corresponding 95% CIs for per decrement of CAG and GGN repeats. We also summarized the MDs in number of repeats between cases and controls. In this meta-analysis, all pooled analyses were performed with random-effects model using the method of DerSimonian-Laird, with the estimate of heterogeneity being taken from the from the Mantel-Haenszel model. Subgroup analyses were also performed according to ethnicity (Caucasian, Asian, Africa, or Hispanic), study design (prospective, i.e. nested or retrospective case-control study), control status, and histology grade of prostate cancer (localized and advanced). In addition, meta-regression analysis was also performed for interaction of between-group. Sensitivity analysis was performed by removing each study at a time. Publication bias was detected using contour-enhanced funnel plot and Egger’s linear regression method. Statistical analysis was performed using Stata 12.0 software. A two-sided P value of 0.05 was used, except for heterogeneity test (0.1).

## Results

### Study characteristics

**Figure 1:**
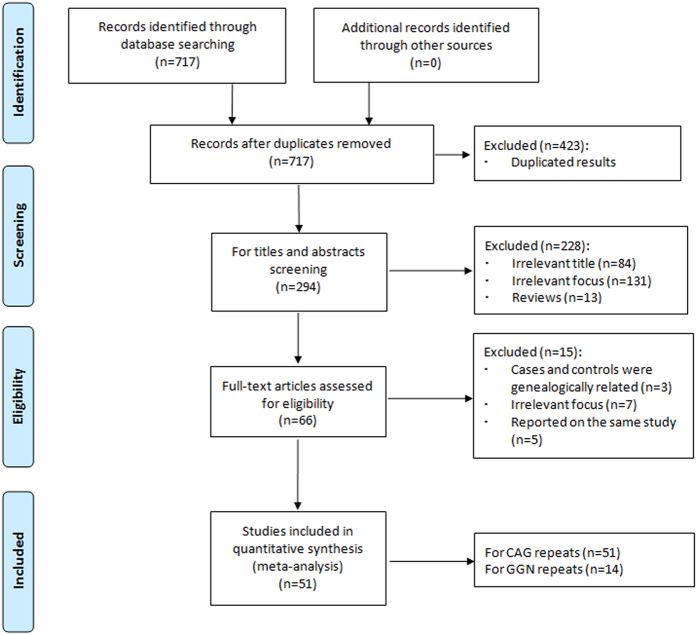
Flow chart for this meta-analysis.

**Figure 2:**
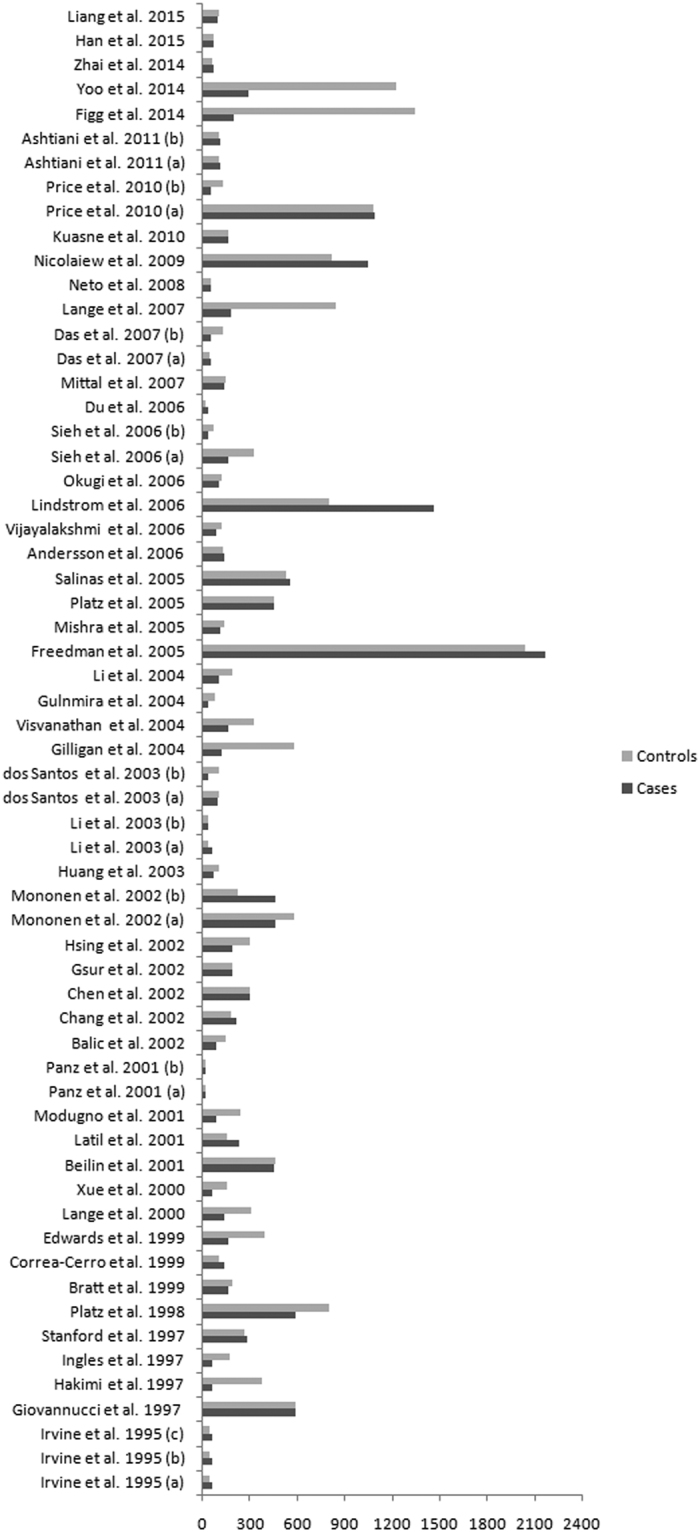
Sample size of the CAG repeat polymorphism.

**Figure 3:**
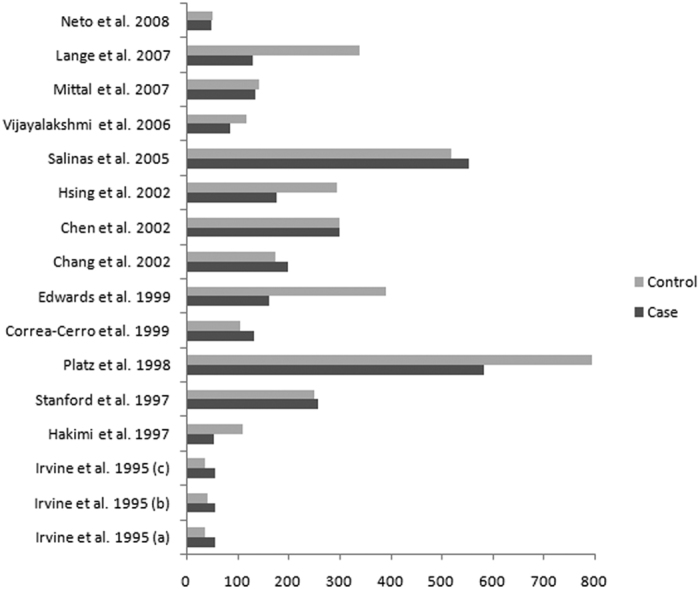
Sample size of the GGN repeat polymorphism.

A total of 717 relevant publications were identified from the electronic literature search. The PRISMA flow diagram was presented in Fig. 1, which shows the detail of inclusion and exclusion of studies. Ultimately, 51 publications^16^,^17^,^18^,^19^,^20^,^21^,^22^,^23^,^24^,^25^,^26^,^27^,^28^,^29^,^30^,^31^,^32^,^33^,^34^,^35^,^36^,^37^,^38^,^39^,^40^,^41^,^42^,^43^,^44^,^45^,^46^,^47^,^48^,^49^,^50^,^51^,^52^,^53^,^54^,^55^,^56^,^57^,^58^,^59^,^60^,^61^,^62^,^63^,^64^,^65^,^66^ were included in the meta-analysis, in which 51 publications^16^,^17^,^18^,^19^,^20^,^21^,^22^,^23^,^24^,^25^,^26^,^27^,^28^,^29^,^30^,^31^,^32^,^33^,^34^,^35^,^36^,^37^,^38^,^39^,^40^,^41^,^42^,^43^,^44^,^45^,^46^,^47^,^48^,^49^,^50^,^51^,^52^,^53^,^54^,^55^,^56^,^57^,^58^,^59^,^60^,^61^,^62^,^63^,^64^,^65^,^66^ with 61 case-control studies (14 803 cases and 18 888 controls, Fig. 2) for CAG repeats and 14 publications^16^,^18^,^20^,^21^,^23^,^24^,^32^,^33^,^35^,^47^,^53^,^54^,^56^,^57^ with 16 case-control studies (2986 cases and 3705 controls, Fig. 3) for GGN repeats. The characteristics of included studies were shown in Table 1.

### Association between CAG repeats polymorphism and prostate cancer risk

**Figure 4:**
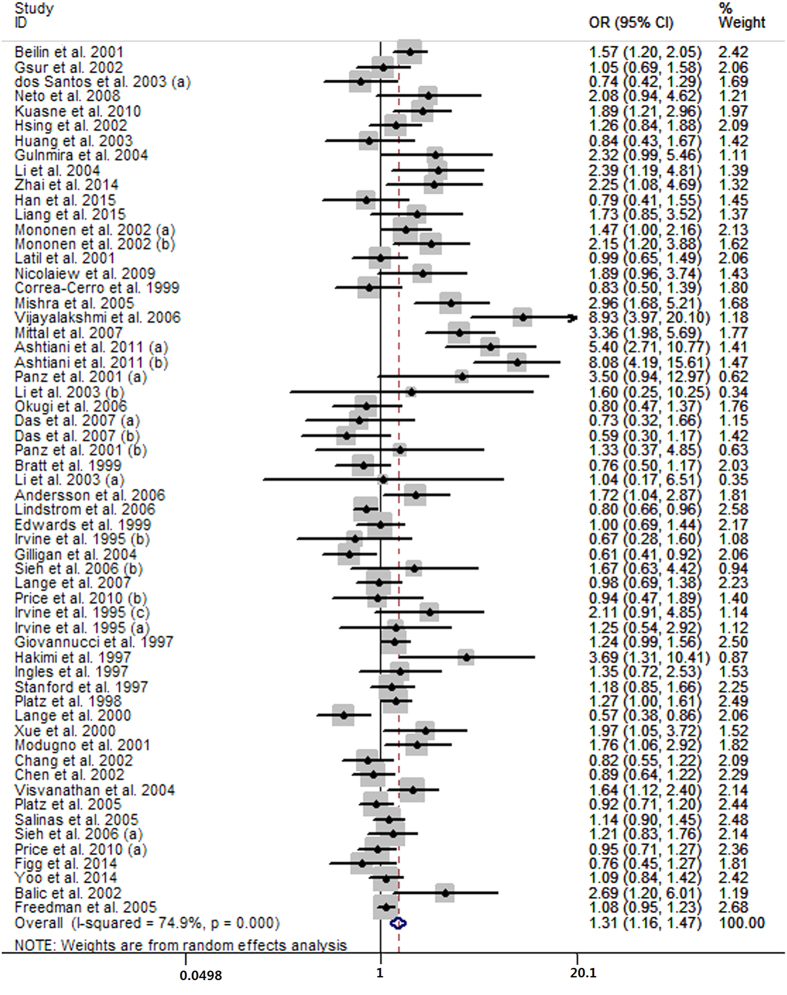
Forest plot of short CAG repeats versus long CAG repeats.

**Figure 5:**
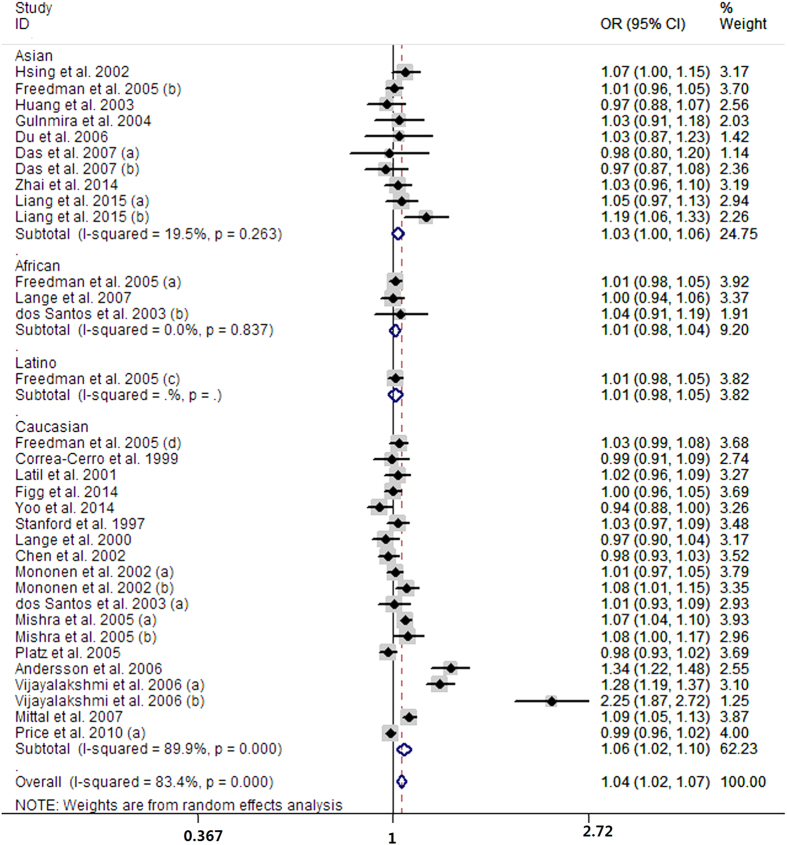
Forest plot of per one CAG repeat decrement and risk of prostate cancer risk.

**Figure 6:**
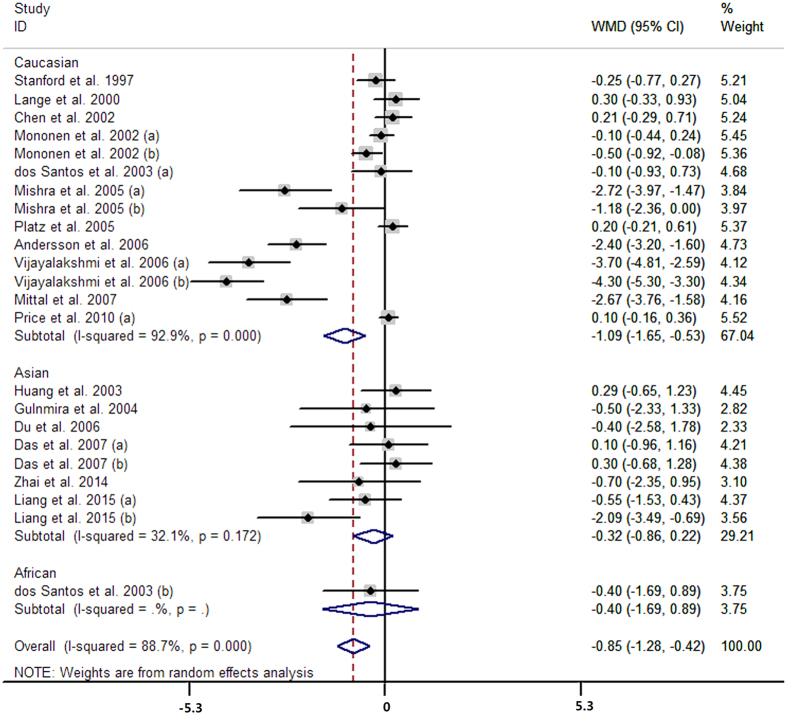
Forest plot of difference in number of CAG repeat length between cases and controls.

Fifty-one case-control studies conveyed data on the short versus long CAG repeats. The pooled analysis showed that men with short CAG repeats carried higher risk of prostate cancer than long CAG repeats (OR = 1.31, 95% CI 1.16 to 1.47; I^2^ = 74.9%, P for heterogeneity <0.01; Fig. 4). Thirty-three case-control studies presented the data for per one CAG decrement and the summarized OR was 1.04 (95% CI 1.02 to 1.07; I^2^ = 83.4%, P for heterogeneity <0.01; Fig. 5) for men with per one CAG decrement. The aggregated analysis suggested that prostate cancer cases seemed to have on average 0.85 fewer CAG repeat length than controls (MD = −0.85, 95% CI −1.28 to −0.42; I^2^ = 88.7%, P for heterogeneity <0.01; Fig. 6) with 23 case-control studies.

### Association between GGN repeats polymorphism and prostate cancer risk

**Figure 7:**
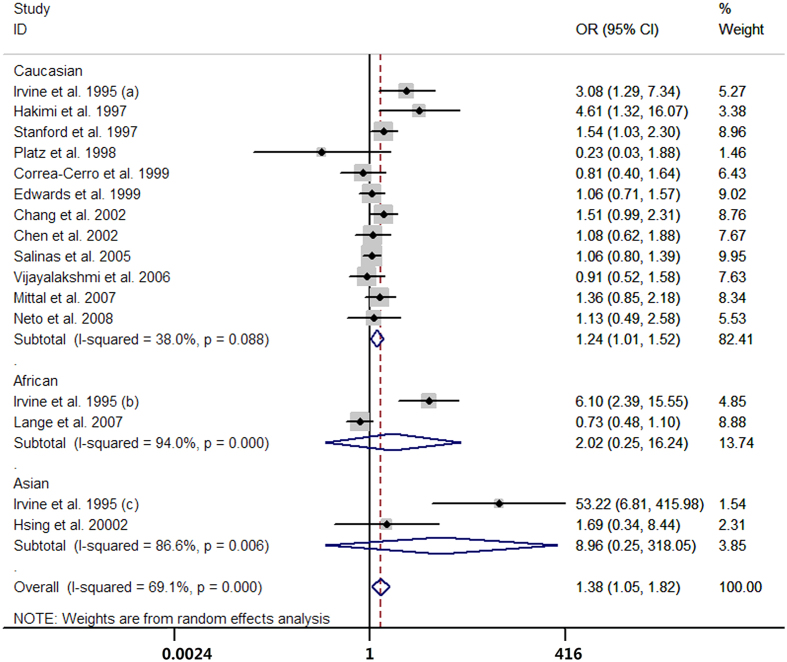
Forest plot of short GGN repeats versus long GGN repeats.

**Figure 8:**
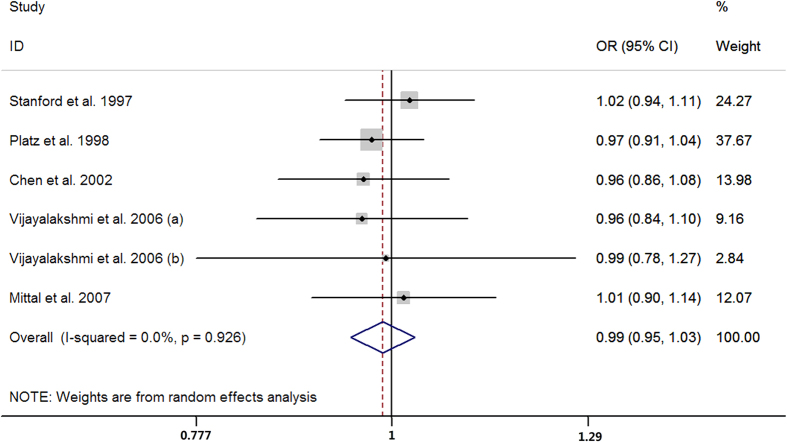
Forest plot of per one GGN repeat decrement and risk of prostate cancer risk.

**Figure 9:**
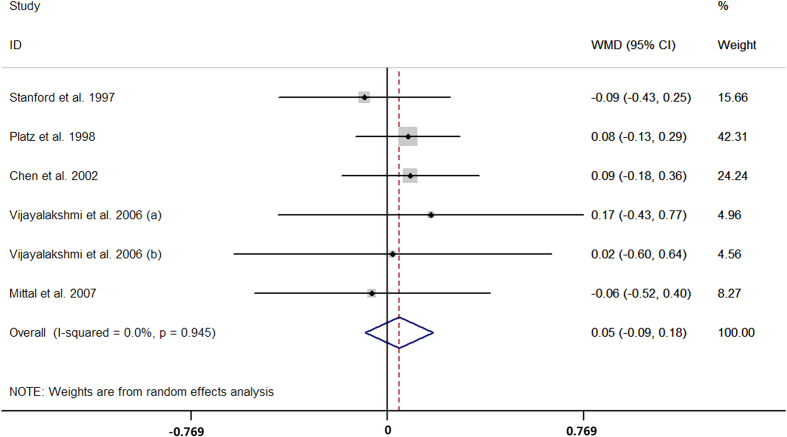
Forest plot of difference in number of GGN repeat length between cases and controls.

Sixteen case-control studies provided data on the short versus long GGN repeats. The pooled results showed that men with short GGN repeats carried higher risk of prostate cancer than long GGN repeats (OR = 1.38, 95% CI 1.05 to 1.82; I^2^ = 69.1%, P for heterogeneity <0.01; Fig. 7). Six case-control studies presented the data for per one GGN decrement and the summarized OR was 0.99 (95% CI 0.95 to 1.03; I^2^ = 0.0%, P for heterogeneity = 0.93; Fig. 8) per GGN. The summarized MD of GGN repeats showed no significant difference between the prostate cancer cases and controls (MD = 0.05, 95% CI −0.09 to 0.18; I^2^ = 0.0%, P for heterogeneity = 0.95; Fig. 9) with six case-control studies.

### Haplotype analysis of CAG and GGN repeat polymorphisms

**Figure 10:**
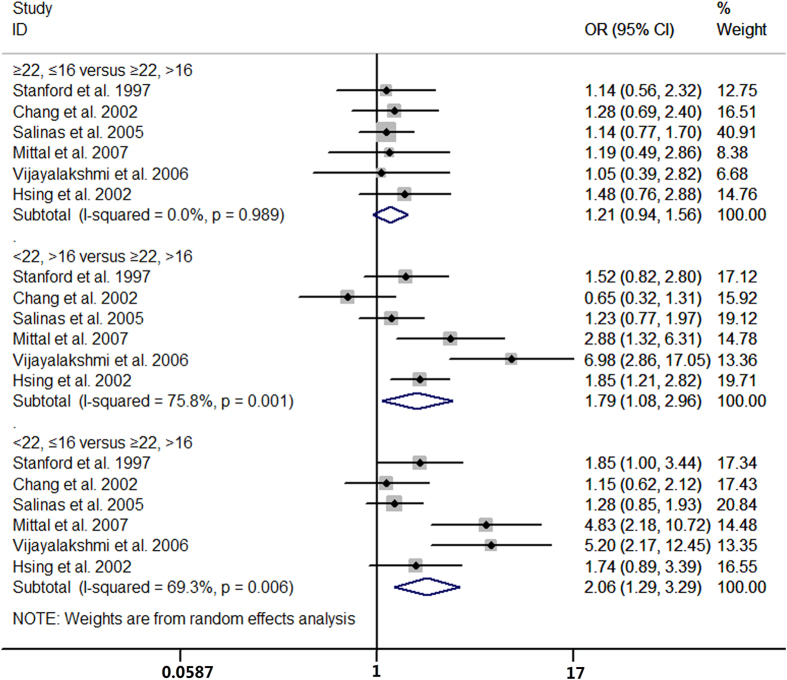
Haplotype analysis of CAG and GGN repeat polymorphisms and risk of prostate cancer.

Six case-control studies provided data for haplotype analysis. The estimated ORs were 2.06 (95% CI 1.29 to 3.29; I^2^ = 69.3%, P for heterogeneity = 0.006), 1.79 (95% CI 1.08 to 2.96; I^2^ = 75.8%, P for heterogeneity = 0.001), and 1.21 (95% CI 0.94 to 1.56; I^2^ = 0, P for heterogeneity = 0.99) for haplotypes CAG <22/GGN ≤16, CAG <22/GGN >16, and CAG ≥22/GGN ≤16 compared with CAG ≥22/GGN >16 (Fig. 10).

### Subgroup, meta-regression and sensitivity analysis

**Figure 11:**
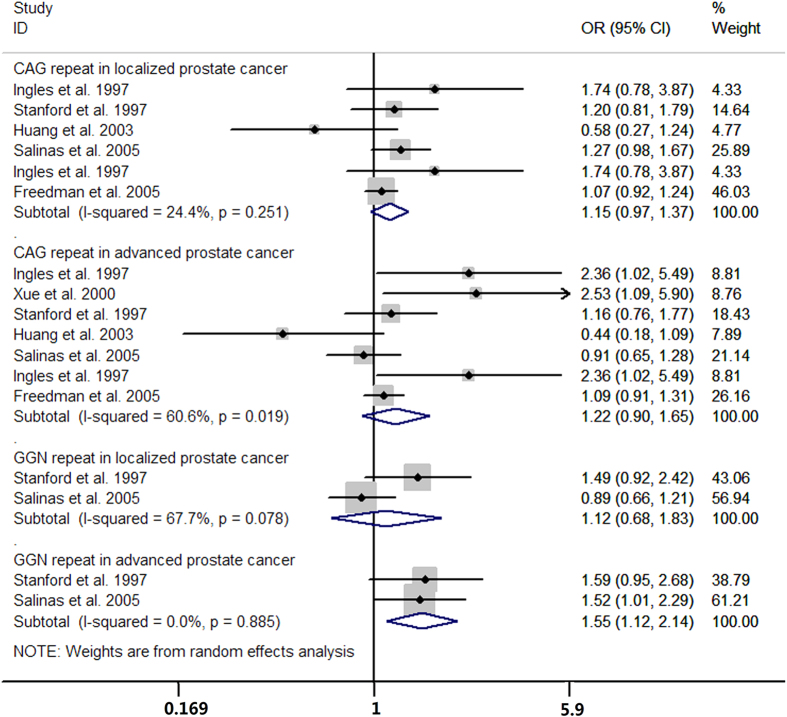
Subgroup analysis of histology grade of prostate cancer.

**Figure 12:**
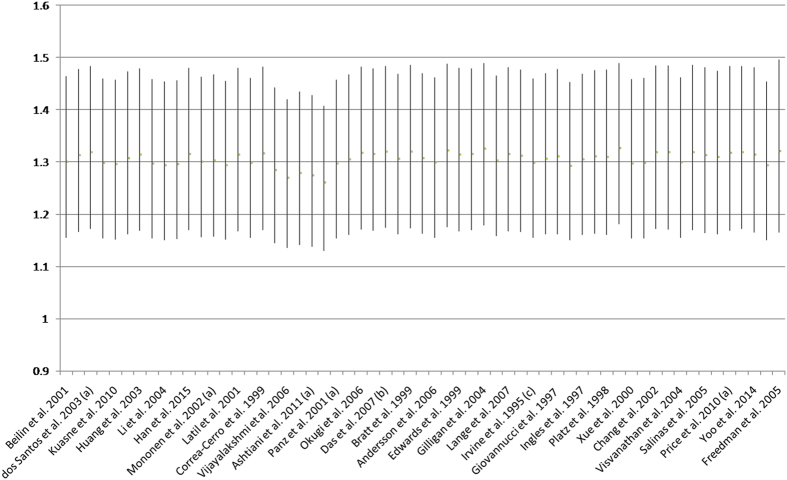
Sensitivity analysis of CAG repeat decrement and risk of prostate cancer risk.

Subgroup analyses were conducted according to ethnicity, study design, control status, and histology grade of prostate cancer. The results of subgroup analyses showed that the elevated risk of prostate cancer in both CAG and GGN repeat polymorphisms were more predominant among Caucasian populations (Tables 2, 3 and 4) and the increased risk of prostate cancer of long GGN repeats were more predominant in advanced prostate cancer cases (Fig. 11). Meta-regression analysis did not detect any significant difference between subgroups (Tables 2, 3 and 4). Subgroup analysis showed that the result of CAG repeat length and risk of prostate cancer was robust (Fig. 12).

### Publication bias

**Figure 13:**
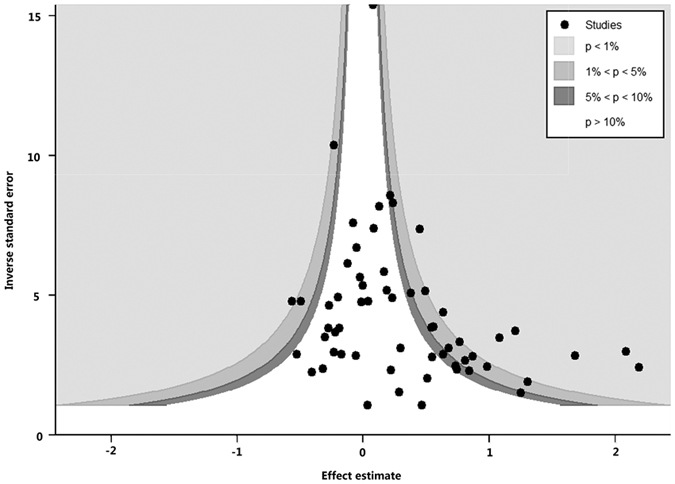
Contour-enhanced funnel plot of CAG repeat polymorphism.

**Figure 14:**
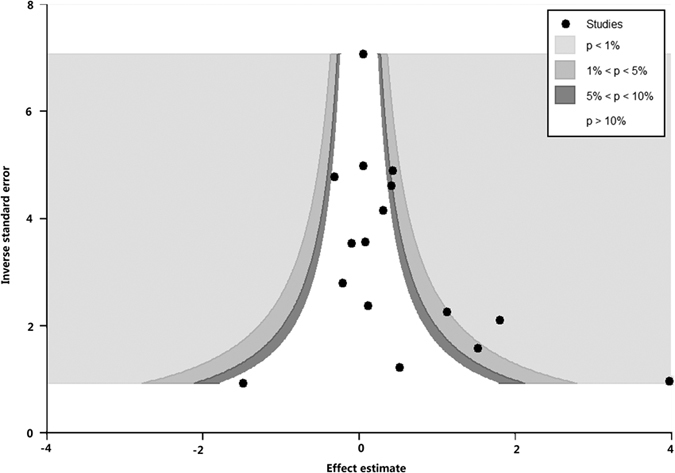
Contour-enhanced funnel plot of GGN repeat polymorphism.

Publication bias was detected using contour-enhanced funnel plot and Egger’s linear regression method. Contour-enhanced funnel plots showed that publication bias might exist for the short versus long CAG repeat polymorphism (Fig. 13) and no publication bias existed in the short versus long GGN repeat polymorphism (Fig. 14). Egger’s linear regression method supported the aforementioned conclusion (P = 0.004 for CAG repeats; P = 0.07 for GGN repeats).

## Discussion

The present meta-analysis summarizes the evidence to date regarding the association between CAG and GGN repeat polymorphisms of androgen receptor and the risk of prostate cancer. The results suggested that short CAG and GGN repeats in the androgen receptor gene were associated with increased risk of prostate cancer, especially in Caucasians.

The short CAG repeats (<22) and short GGN repeats (≤16) carry a roughly 1.31- and 1.38-fold higher risk of developing prostate cancer compared with subjects with long CAG (≥22) repeats and long GGN repeats (>16), respectively. Each decrement in CAG repeat presented 1.04-fold higher risk of developing prostate cancer. Prostate cancer cases presented an average 0.85 fewer CAG repeats than controls. In Caucasians, the aforementioned elevated risk was increased. This could be due to that more studies conducted in Caucasians, which provided greater statistical power for detecting small gene effect. Specifically, the prostate cancer cases in Caucasian population carried an average 1.09 fewer CAG repeats than controls. This difference might yield certain measurable biological impact in prostate carcinogenesis, such as early diagnosis and gene therapy.

An interaction between CAG and GGN repeat polymorphisms in increasing the prostate cancer susceptibility was documented by our meta-analysis. Haplotype analysis showed that short CAG and short GGN repeats carriers presented 2.06-fold higher risk of developing prostate cancer compared with long CAG and long GGN repeats carriers. Moreover, the short CAG repeats and long GGN repeats carriers presented 1.79-fold higher risk of developing prostate cancer compared with long CAG and long GGN repeats carriers.

In 2004, Zeegers *et al*.^6^ published the first meta-analysis regarding the association between CAG and GGN repeat length polymorphisms in the androgen receptor gene and prostate cancer risk, in which included 23 articles with 19 retrospective case-control studies and 5 prospective case-control studies, comprising a total of 4274 cases and 5275 controls. They found that the presence of shorter repeats seemed to be modestly associated with prostate cancer risk. However, they did not found any significant difference in number of repeats between cases and controls. In 2012, Gu *et al*.^10^ aggregated 27 articles to evaluate the relationship between CAG repeat polymorphism and prostate cancer risk. Their meta-analysis demonstrated that the CAG repeat polymorphism in androgen receptor gene with more than 20 repeats might confer a protective effect among the prostate cancer cases among men 45 years or older only. In 2013, Sun *et al*.^11^ carried out another meta-analysis regarding the association between CAG repeat polymorphism and prostate cancer risk, which included 47 studies with 13 346 cases and 15 172 controls. They suggested that a short CAG repeat polymorphism might increase the risk of prostate cancer compared with the longer CAG repeat, especially in Caucasians and Asians. Compared with the previous meta-analysis^6^,^10^,^11^, our meta-analysis was more comprehensively searched and our meta-analysis included 51 case-control studies (14 803 cases and 18 888 controls) for CAG repeats and 16 case-control studies (2986 cases and 3705 controls) for GGN repeats. In addition, our meta-analysis performed haplotype analysis and suggested that there exists an interaction between CAG and GGN repeat polymorphisms in increasing the prostate cancer susceptibility. Moreover, we found a significant difference in number of CAG repeat length between cases and controls, and the absolute difference in more than 1 repeat in Caucasians.

The present retrospective analysis has some limitations. First, the evidence of between study heterogeneity was apparent, and the heterogeneity might distort the conclusion of the current meta-analysis^67^,^68^,^69^. Additionally, the meta-regression analysis failed to identify the source of heterogeneity. Second, the standard of cutpoint of repeat length polymorphisms varied in different studies. This might in part contribute to the between study heterogeneity. Third, the screening policy of prostate cancer also varies between countries. Especially in United States, the prostate-specific antigen screening of the general population is more commonly used than other countries^6^. These different screening policies might also be responsible for the between study heterogeneity. Fourth, the publication bias was detected in the present meta-analysis for the association between CAG repeat polymorphism and risk of prostate cancer. The existing publication bias indicated that certain studies with negative results for the association between CAG repeat polymorphism and prostate cancer risk are under-represented in the literature. The publication bias also might distort the conclusion of the present meta-analysis. Ultimately, the meta-analysis is a secondary analysis; therefore, we could not handle the problem of between study heterogeneity.

In summary, our meta-analysis indicated that short CAG and GGN repeats in androgen receptor gene were associated with increased risk of prostate cancer, especially in Caucasians.

## Additional Information

**How to cite this article**: Weng, H. *et al*. Androgen receptor gene polymorphisms and risk of prostate cancer: a meta-analysis. *Sci. Rep.* **7**, 40554; doi: 10.1038/srep40554 (2017).

**Publisher's note:** Springer Nature remains neutral with regard to jurisdictional claims in published maps and institutional affiliations.

## Figures and Tables

**Table 1:** Characteristics of included studies in the meta-analysis.

Reference	Country	Race	Study design	Control status	Age, yr (ca/co)	Advanced cases (%)	Sample size		Repeat cutpoint
							Cases	Controls	
**CAG repeats**									
Irvine *et al*. 1995 (a)	US	Caucasian	Retrospective	Healthy	57.8/NR	46	57	39	22
Irvine *et al*. 1995 (b)	US	African	Retrospective	Healthy	57.8/NR	46	57	44	22
Irvine *et al*. 1995 (c)	US	Asian	Retrospective	Healthy	57.8/NR	46	57	39	22
Giovannucci *et al*. 1997	US	Caucasian	Prospective	Healthy	NR	30.7	587	588	22
Hakimi *et al*. 1997	US	Caucasian	Retrospective	Healthy	62.1/NR	42.4	59	370	17
Ingles *et al*. 1997	US	Caucasian	Retrospective	Healthy	57.8/NR	46	57	169	22
Stanford *et al*. 1997	US	Caucasian	Retrospective	Healthy	54.9/54	45.9	281	266	22
Platz *et al*. 1998	US	Caucasian	Prospective	Healthy	62/NR	46.6	582	794	23
Bratt *et al*. 1999	Sweden	Caucasian	Retrospective	Healthy	70.2/NR	1.6	160	186	22
Correa-Cerro *et al*. 1999	Germany	Caucasian	Retrospective	Healthy	68.2/71.2	NR	132	105	22
Edwards *et al*. 1999	UK	Caucasian	Retrospective	Healthy	68.1/NR	75.3	162	390	22
Lange *et al*. 2000	US	Caucasian	Retrospective	Healthy	64/NR	NR	133	305	22
Xue *et al*. 2000	US	Caucasian	Retrospective	Healthy	57.8/58.2	46	57	156	20
Beilin *et al*. 2001	Australia	Caucasian	Retrospective	Healthy	67/66.6	39.2	448	456	22
Latil *et al*. 2001	France	Caucasian	Prospective	Healthy	70.5/71.7	69.8	226	156	23
Modugno *et al*. 2001	US	Caucasian	Retrospective	Healthy	68.9/73.6	NR	88	241	23
Panz *et al*. 2001 (a)	Israel	Caucasian	Retrospective	Healthy	76/NR	30	20	20	22
Panz *et al*. 2001 (b)	South Africa	African	Retrospective	Healthy	68/NR	30	20	20	22
Balic *et al*. 2002	US	Hispanic	Retrospective	Healthy	64/57	NR	82	145	18
Chang *et al*. 2002	US	Caucasian	Retrospective	Healthy	60.9/58	NR	210	180	22
Chen *et al*. 2002	US	Caucasian	Prospective	Healthy	61.2/60.8	11.5	300	300	22
Gsur *et al*. 2002	Australia	Caucasian	Retrospective	BPH	65.9/66.5	NR	190	190	22
Hsing *et al*. 2002	China	Asian	Retrospective	Healthy	72.2/71.9	62.6	190	300	22
Mononen *et al*. 2002 (a)	Finland	Caucasian	Retrospective	Healthy	68.1/NR	48.1	461	574	18
Mononen *et al*. 2002 (b)	Finland	Caucasian	Retrospective	BPH	68.1/NR	48.1	461	223	18
Huang *et al*. 2003	China	Asian	Retrospective	Healthy	71.5/71.7	40.9	66	104	22
Li *et al*. 2003 (a)	Sweden	Caucasian	Retrospective	BPH	69/67	NR	59	38	22
Li *et al*. 2003 (b)	Japan	Asian	Retrospective	BPH	71/NR	NR	34	33	22
dos Santos *et al*. 2003 (a)	Brazil	Caucasian	Retrospective	Healthy	65/58	NR	97	100	21
dos Santos *et al*. 2003 (b)	Brazil	African	Retrospective	Healthy	65/58	NR	32	100	NR
Gilligan *et al*. 2004	US	African	Retrospective	Healthy	66.7/55.5	24.5	118	576	22
Visvanathan *et al*. 2004	US	Caucasian	Prospective	Healthy	66.1/66	45.8	164	324	22
Gulnmira *et al*. 2004	China	Asian	Retrospective	Healthy	67.5/66.3	NR	31	80	22
Li *et al*. 2004	China	Asian	Retrospective	Healthy	67.9/67.1	60	105	190	22
Freedman *et al*. 2005	US	Mixed	Prospective	Healthy	45-75	NR	2160	2036	22
Mishra *et al*. 2005	India	Caucasian	Retrospective	Healthy	65.6/63.7	NR	113	133	22
Platz *et al*. 2005	US	Caucasian	Prospective	Healthy	NR	NR	448	448	22
Salinas *et al*. 2005	US	Caucasian	Retrospective	Healthy	NR	33.8	553	523	22
Andersson *et al*. 2006	Sweden	Caucasian	Retrospective	Healthy	76.2/NR	NR	137	125	23
Vijayalakshmi *et al*. 2006	India	Caucasian	Retrospective	Mixed#	67.5/66	NR	87	120	22
Lindstrom *et al*. 2006	Sweden	Caucasian	Retrospective	Healthy	NR	48	1461	796	22
Okugi *et al*. 2006	Japan	Asian	Retrospective	Healthy	69.9/71	NR	102	117	22
Sieh *et al*. 2006 (a)	US	Caucasian	Prospective	Healthy	77.1/NR	31.9	160	320	22
Sieh *et al*. 2006 (b)	US	African	Prospective	Healthy	74.9/NR	45.5	33	71	22
Du *et al*. 2006	China	Asian	Retrospective	Healthy	NR	NR	35	15	NR
Mittal *et al*. 2007	India	Caucasian	Retrospective	Healthy	66.2/64.1	NR	135	142	22
Das *et al*. 2007 (a)	Singapore	Asian	Retrospective	Healthy	66/69	NR	47	46	22
Das *et al*. 2007 (b)	Singapore	Asian	Retrospective	BPH	66/67	NR	47	130	22
Lange *et al*. 2007	US	African	Retrospective	Healthy	40–79	NR	180	840	22
Neto *et al*. 2008	Brazil	Caucasian	Retrospective	Healthy	64/59	NR	49	51	21
Nicolaiew *et al*. 2009	France	Caucasian	Retrospective	Healthy	67/63	NR	1045	814	17
Kuasne *et al*. 2010	Brazil	Caucasian	Retrospective	Healthy	65.3/63.8	38.8	160	160	20
Price *et al*. 2010 (a)	US	Caucasian	Prospective	Healthy	63.4/63.6	NR	1082	1080	19
Price *et al*. 2010 (b)	US	African	Prospective	Healthy	63.4/63.6	NR	47	128	19
Ashtiani *et al*. 2011 (a)	Iran	Caucasian	Retrospective	Healthy	NR	NR	110	100	21
Ashtiani *et al*. 2011 (b)	Iran	Caucasian	Retrospective	BPH	NR	NR	110	99	21
Figg *et al*. 2014	US	Caucasian	Prospective	Healthy	60.4/NR	NR	195	1344	19
Yoo *et al*. 2014	US	Caucasian	Prospective	Healthy	66/63.2	7.9	291	1221	22
Zhai *et al*. 2014	China	Asian	Retrospective	Healthy	67.4/67.9	38.2	68	60	22
Han *et al*. 2015	China	Asian	Retrospective	Healthy	NR	NR	70	70	18
Liang *et al*. 2015	China	Asian	Retrospective	Healthy	64/58	NR	95	98	22
**GGN repeats**									
Irvine *et al*. 1995 (a)	US	Caucasian	Retrospective	Healthy	57.8/NR	46	57	37	16
Irvine *et al*. 1995 (b)	US	African	Retrospective	Healthy	57.8/NR	46	57	41	16
Irvine *et al*. 1995 (c)	US	Asian	Retrospective	Healthy	57.8/NR	46	57	37	16
Hakimi *et al*. 1997	US	Caucasian	Retrospective	Healthy	62.1/NR	42.4	54	110	14
Stanford *et al*. 1997	US	Caucasian	Retrospective	Healthy	54.9/54	45.9	257	250	16
Platz *et al*. 1998	US	Caucasian	Prospective	Healthy	62/NR	46.6	582	794	16
Correa-Cerro *et al*. 1999	Germany	Caucasian	Retrospective	Healthy	68.2/71.2	NR	132	105	16
Edwards *et al*. 1999	UK	Caucasian	Retrospective	Healthy	68.1/NR	75.3	162	390	16
Chang *et al*. 2002	US	Caucasian	Retrospective	Healthy	60.9/58	NR	198	174	16
Chen *et al*. 2002	US	Caucasian	Prospective	Healthy	61.2/60.8	11.5	300	300	16
Hsing *et al*. 2002	China	Asian	Retrospective	Healthy	72.2/71.9	62.6	178	295	16
Salinas *et al*. 2005	US	Caucasian	Retrospective	Healthy	40–64	33.8	553	520	16
Vijayalakshmi *et al*. 2006	India	Caucasian	Retrospective	Mixed	67.5/66	NR	86	119	21
Mittal *et al*. 2007	India	Caucasian	Retrospective	Healthy	66.2/64.1	NR	135	142	22
Lange *et al*. 2007	US	African	Retrospective	Healthy	40–79	NR	129	340	16
Neto *et al*. 2008	Brazil	Caucasian	Retrospective	Healthy	64/59	NR	49	51	17

NR, not report.

**Table 2:** The results of overall and subgroup analyses of the association between CAG repeats and prostate cancer risk.

	No. studies	OR (95% CI)	P_OR_	I^2^	P_heterogeneity_	P_interaction_
**Short versus long**	59	1.31 (1.16 to 1.47)	<0.01	74.9	<0.01	
Ethnicity						0.07
Caucasian	39	1.39 (1.20 to 1.61)	<0.01	80.1	<0.01	
Asian	12	1.24 (0.93 to 1.65)	0.15	50	0.02	
African	6	0.86 (0.66 to 1.12)	0.26	13.5	0.33	
Hispanic	1	2.69 (1.20 to 6.01)	0.02	NA	NA	
Study design						0.05
Retrospective	46	1.43 (1.21 to 1.70)	<0.01	78.9	<0.01	
Prospective	13	1.09 (1.00 to 1.20)	0.06	17.2	0.27	
Control status						0.58
Healthy	52	1.23 (1.11 to 1.37)	<0.01	69	<0.01	
BPH	6	1.68 (0.73 to 3.87)	0.23	86.4	<0.01	
**Increment per repeat**	33	1.04 (1.02 to 1.07)	<0.01	83.4	<0.01	
Ethnicity						0.41
Caucasian	19	1.06 (1.02 to 1.10)	<0.01	89.9	<0.01	
Asian	10	1.03 (1.00 to 1.06)	0.06	19.5	0.26	
African	3	1.01 (0.98 to 1.04)	0.39	0	0.84	
Latino	1	1.01 (0.98 to 1.05)	0.44	NA	NA	
Study design						0.16
Retrospective	23	1.08 (1.04 to 1.12)	<0.01	84.6	<0.01	
Prospective	10	1.01 (0.99 to 1.01)	0.98	9.6	0.35	
Control status						0.14
Healthy	29	1.03 (1.01 to 1.06)	<0.01	76.8	<0.01	
BPH	4	1.24 (1.00 to 1.53)	0.05	95.1	<0.01	

BPH, benign prostatic hyperplasia; OR, odds ratio; CI, confidence interval; NA, not available.

**Table 3:** Results of length of CAG repeats and risk of prostate cancer.

	No. studies	MD (95% CI)	P_MD_	I^2^	P_heterogeneity_	P_interaction_
**Length of CAG repeat**	23	−0.85 (−1.28 to −0.42)	<0.01	88.7	<0.01	
Ethnicity						0.11
Caucasian	14	−1.09 (−1.65 to −0.53)	<0.01	92.9	<0.01	
Asian	8	−0.32 (−0.86 to 0.22)	0.25	32.1	0.17	
African	1	−0.40 (−1.69 to 0.89)	0.55	NA	NA	
Study design						0.07
Retrospective	20	−1.06 (−1.60 to −0.51)	<0.01	88.2	<0.01	
Prospective	3	0.14 (−0.06 to 0.34)	0.17	0.0	0.88	
Control status						0.81
Healthy	19	−0.72 (−1.15 to −0.29)	<0.01	85.8	<0.01	
BPH	4	−1.40 (−3.19 to 0.38)	0.12	94.5	<0.01	

BPH, benign prostatic hyperplasia; MD, mead difference; CI, confidence interval; NA, not available.

**Table 4:** Results of the association between GGN repeats and prostate cancer.

	No. studies	OR (95% CI)	P_OR_	I^2^	P_heterogeneity_	P_interaction_
**Short versus long**	16	1.38 (1.05 to 1.82)	0.02	69.1	<0.01	
Ethnicity						0.52
Caucasian	12	1.24 (1.01 to 1.52)	0.04	38	0.09	
Asian	2	8.96 (0.25 to 318.05)	0.51	86.6	0.01	
African	2	2.02 (0.25 to 16.24)	0.23	94	<0.01	
Study design						0.37
Retrospective	14	1.46 (1.09 to 1.97)	0.01	71.8	<0.01	
Prospective	2	0.70 (0.17 to 2.80)	0.61	49.9	0.16	
Control status						0.57
Healthy	15	1.44 (1.07 to 1.93)	0.02	70.5	<0.01	
Mixed	1	0.91 (0.52 to 1.58)	0.73	NA	NA	

OR, odds ratio; CI, confidence interval; NA, not available.
